# Kaposi sarcoma in unusual locations

**DOI:** 10.1186/1471-2407-8-190

**Published:** 2008-07-07

**Authors:** Liron Pantanowitz, Bruce J Dezube

**Affiliations:** 1 Department of Pathology, Baystate Medical Center, Tufts University School of Medicine, Springfield, MA, USA; 2 Department of Medicine, Division of Hematology Oncology, Beth Israel Deaconess Medical Center, Harvard Medical School, Boston, MA, USA

## Abstract

Kaposi sarcoma (KS) is a multifocal, vascular lesion of low-grade malignant potential that presents most frequently in mucocutaneous sites. KS also commonly involves lymph nodes and visceral organs. This article deals with the manifestation of KS in unusual anatomic regions. Unusual locations of KS involvement include the musculoskeletal system, central and peripheral nervous system, larynx, eye, major salivary glands, endocrine organs, heart, thoracic duct, urinary system and breast. The development of KS within wounds and blood clots is also presented. KS in these atypical sites may prove difficult to diagnose, resulting in patient mismanagement. Theories to explain the rarity and development of KS in these unusual sites are discussed.

## Background

Kaposi sarcoma (KS) is a vascular lesion of low-grade malignant potential that is associated with Human Herpesvirus-8 (HHV8) infection. KS is a multifocal tumor that manifests most frequently in mucocutaneous sites, typically the skin of the lower extremities, face, trunk, genitalia and oropharyngeal mucosa (Table 1). KS also commonly involves lymph nodes and visceral organs, most notably the respiratory and gastrointestinal tracts. Peculiar presentations of KS reported in relation to the gastrointestinal tract involvement include primary KS of the appendix [1], isolated rectal KS [2], and KS with mesenteric localization [3]. Scores of authors have reported on the occurrence of KS in numerous unusual sites (i.e. anatomic locations other than the aforementioned sites). This review discusses the manifestation of KS in several of these unusual regions of the human body, and highlights what can be learned from these perceptive published observations.

**Table 1:** Usual and unusual anatomical locations of Kaposi sarcoma

**Usual Locations**	**Unusual Locations**
• Skin	• Bones & skeletal muscles
• Oral mucosa	• Peripheral nerves
• Lymph nodes (superficial & deep)	• Brain & spinal cord
• Lungs, endobronchial tract, & pleura	• Larynx
• Gastrointestinal tract	• Eye & ear
• External genitalia	• Major salivary glands
• Oropharynx	• Adrenal & thyroid gland
• Tonsils	• Heart
• Nasal cavity	• Thoracic duct
• Liver	• Kidney
• Spleen	• Ureter & urinary bladder
• Bone marrow	• Breast
	• Gonads
	• Pancreas
	• Wounds & blood clots

## Musculoskeletal system

KS involvement of the musculoskeletal system is a rare occurrence [4]. Nevertheless, around 70 such cases have been reported. Dr. Moriz Kaposi was the first to document KS involvement of the skeletal system. He described ulcerating KS lesions on the extremities of his patients that penetrated into underlying bone [5]. Limited early literature, pre-dating the AIDS epidemic, reported that skeletal involvement was a frequent (~20%) finding in African (endemic) KS [6-8]. The largest review to date on KS involvement of the musculoskeletal system included 66 previously published cases reported from 1925 to 2006 [9]. Only three cases in this review included KS within skeletal muscle, all AIDS-related KS, that had infiltrated the gastrocnemius muscle [10], intercostal muscles with associated rib destruction [11], and the masseter muscle in association with oral KS [12]. Within the musculoskeletal system, osseous KS lesions are more frequently encountered than KS in skeletal muscle. Involvement of bone marrow, without causing an osseous lesion, is far more common in AIDS-related KS [13-16]. In a series of 45 HIV-positive patients manifesting with musculoskeletal abnormalities, researchers found that only two individuals had KS of the bone [17]. Most of the patients in this series had infections and several had bone lymphomas.

The musculoskeletal system has been shown to be involved by all KS epidemiological forms, including AIDS-related, Classic, African (endemic), and infrequently transplanted-associated KS [9,18-21]. Reports of African KS bone lesions originated mainly from central Africa (e.g. Uganda), but also from Northern African countries (e.g. Algeria, Morocco) and from South Africa. Despite an extensive literature search, we were able to identify only a single case of transplanted-associated KS involving bone. This particular 40 year-old female transplant recipient, who was being treated with cyclosporine, died 8.3 months after her kidney transplant of disseminated KS, diagnosed only by post-mortem examination [22].

African and Classic KS lesions tend to involve the peripheral skeleton, whereas AIDS-related KS more commonly involves the axial skeleton (vertebrae, ribs, sternum, and pelvis) and/or maxillofacial bones [9,23-32]. KS of the skull has been noted in the parietal and temporal bones, paranasal sinus, maxilla, hard palate and mandible. Other bones reported to be involved by KS include the vertebrae (T11 to L4), ribs, sternum, pelvis, long bones of the extremities (humerus, radius, ulna, femur, tibia, fibula), as well as those of the hands (metacarpals) and feet (talus, calcaneus, and the 3^rd^, 4^th ^and 5^th ^metatarsals). Asymmetric bone involvement by KS appears to be the rule. Joint involvement is unusual, but has been reported [33].

Osseous KS lesions are more likely to be associated with long-standing and locally aggressive cutaneous African and Classic KS lesions, or with AIDS-associated mucosal KS lesions that penetrate underlying bone [9]. Patients with osseous KS may complain of bone pain with limited mobility. Serious sequelae have occurred, such as acute spinal cord compression [34,35]. KS of the jaw bones can cause pain, headache, increased tooth mobility, paresthesia, or can present with an intraoral tumor mass. Infrequently, osseous KS lesions may be asymptomatic and remain undetected, or may be discovered only incidentally or at autopsy. Pathological fractures in afflicted patients do not seem to be a problem. KS involvement of bone without KS disease elsewhere is exceptional [9]. Patients with osseous KS usually have concomitant non-osseous KS lesions, particularly those with AIDS. Very few cases, all AIDS patients, have had primary intraosseous KS [25,36,37]. Most of the cases reported in the literature to date were due to KS arising in overlying skin or mucosa, in which KS eroded into underlying bone [4,9]. Those with AIDS-associated osseous KS often present with a CD4+ T-cell count of <100 cells/mm^3^[9]. AIDS-related KS that involves bone usually portends an unfavorable prognosis [9,38].

Most osseous KS lesions are osteolytic, with cortical and at times almost complete bone destruction (osteolysis). KS of the bone does not present well on plain radiographs, despite their frequent osteolytic nature [38-40]. CT scan and MRI appear to be superior for the detection of KS bone lesions [9,11]. Biopsy of a suspected KS lesion is essential, because other conditions such as bacillary angiomatosis can present with similar clinical and radiological findings [41]. Radiation therapy has provided patients with prompt relief of bone pain [13,21,31]. Treatment of osseous KS whether by surgery, chemotherapy and/or radiation seems to have limited success [1,9]. Nevertheless, individual case reports describing improvement of patients following radiation and/or systemic chemotherapy have been published [26,36,42]. Patient's response to therapy requires monitoring radiological changes in addition to routine clinical observation. With intraosseous KS involvement, successful management generally requires a multidisciplinary approach [43].

## Nervous system

### 1. Peripheral nervous system

Peripheral nerve involvement by KS is rare. In a postmortem study of temporal bones obtained from patients with AIDS, investigators discovered KS in the eighth cranial nerve of one patient [44]. An autopsy in this individual revealed concomitant KS of the skin and lungs. In another study of African KS cases, KS was portrayed growing along perineural spaces of large nerves [45]. This paper also described small nerves encased in subcutaneous KS nodules. The only case with documented peripheral nervous system involvement is of a 75-year-old woman who presented with low back and leg pain, asymmetrical lower leg weakness, muscle wasting, paresthesiae, and bilateral lower extremity edema [46]. At autopsy, KS was identified infiltrating the lumbar spinal cord, sacral plexus, sciatic and femoral nerves of this patient. In the spinal cord there was nearby recent microinfarction, and petechial hemorrhage was noted. Along involved peripheral nerves there was associated endoneurial edema and massive destruction of axons and myelin sheaths. Two lumbar dorsal root ganglia contained KS tumor in their capsules. Sections taken from distal popliteal nerves showed wallerian degeneration. Skeletal muscle from the lower extremities in this individual revealed neurogenic atrophy.

### 2. Central nervous system (CNS)

#### 2.1 Brain

Around 15 cases of suspected brain involvement with KS have been reported [47-58], but in only a few of these cases did an autopsy actually confirm KS within intracranial lesions [50,52,55]. Human herpes virus-8 (HHV8) DNA was detected by PCR in one patient's intracranial lesion [58]. In a review of a transplant tumor registry in Israel, of 29 donors with CNS tumors 7% were due to KS [59]. Patients with Classic, African, AIDS-related and transplant-associated KS epidemiological forms involving the brain have all been reported. KS has been documented predominantly in the cerebrum, but has been noted also in the cerebellum, pons, meninges and dura mater. KS within the brain appears to almost always occur with generalized KS disease, including widespread visceral involvement. Symptoms related to brain KS are not well reported. One patient (16-year-old boy) with transplant-associated intracranial KS presented with a generalized tonic and clonic seizure [58]. Another patient with African KS developed hemiparesis and urinary incontinence due to widespread KS involvement of her skull, vertebrae and brain [50]. At autopsy, this patient's brain lesions included five well-delineated nodules of KS, showing considerable hemorrhage and necrosis. KS tumor cells in these nodules were reported to be markedly pleomorphic.

A few of the cases reported have been regarded with some suspicion by others, as the patients in these reports did not have skin lesions [60]. Based upon available information, KS brain lesions have ranged in size from 2 mm to large (up to 2 cm) destructive deposits. The radiologic appearance of intracranial KS has only been rarely described [52,61]. On CT scan, KS appears as homogeneous, hyperdense lesions with little surrounding edema and minimal mass effect. On MRI, brain KS lesions also appear as a homogeneous mass, of high signal intensity with a relatively T2 weighted sequence. In one case the CT scan (without contrast material) was negative, while the MR image showed an abnormal focus.

#### 2.2 Spinal cord

There have been two cases of KS infiltrating the spinal cord [34,48]. In one patient presenting acutely with spinal cord compression, there was concomitant destructive KS of the thoracic and lumbar vertebrae [34]. In the other patient with chronic neurological symptoms, KS involved the lumbar spinal cord [48]. Autopsy in this latter individual also revealed nearby recent microinfarction and petechial hemorrhages in the spinal cord.

## Larynx

Head and neck involvement of KS is not unusual. However, laryngeal involvement is somewhat of an infrequent manifestation. There have been approximately 25 accounts of KS of the larynx [62-73]. Most patients have had AIDS-related KS, although HIV-negative persons with laryngeal KS have also been noted [72,74]. Of the AIDS cases reported, the majority (91%) were males of mean age 35 years (range 24–56 years), with advanced HIV disease, that were antiretroviral naïve [73]. This may explain why many afflicted persons also had oropharyngeal, as well as cutaneous and visceral KS.

Presenting symptoms may include hoarseness, throat discomfort, urge to cough, aphonia, dysphagia, stridor or complete airway obstruction. Examination may reveal laryngeal edema or more likely a purple vascular mass lesion. The lesion's surface can appear verrucous in nature, due to deposits of dry secretion [62]. The diagnosis can be established by laryngoscopy or radiologic studies. A CT scan of the larynx may help delineate laryngeal mass lesions. While a diagnostic biopsy in several patients was performed without complications, biopsy of such vascular laryngeal lesions has been associated with brisk and potentially fatal bleeding [65].

Therapeutic options for this scenario include low-dose local irradiation, intralesional chemotherapy or laser ablation, and systemic therapy, particularly if there is disseminated KS. For laryngeal KS lesions producing acute or impending airway obstruction urgent intervention is necessary. However, depending on the location of the KS lesion, tracheostomy may contribute to mortality as a result of fatal hemorrhage [66]. Therefore, cricothyrotomy has been recommended by some authors as an alternative approach to life-threatening emergencies in this setting.

## Eye

Periorbital edema may occur with KS of the face [75,76]. External ocular KS lesions are also not uncommon. KS of the conjunctiva and ocular adnexa has been reported in association with Classic and AIDS-related KS [77-87]. Isolated bulbar conjunctival and eyelid KS has been noted [88,89]. In a study of 6,552 AIDS patients from Buenos Aires in Argentina, ocular KS was diagnosed in 17 (0.25%) individuals [90]. Lesions in this study predominated in eyelids, most with the inferior eyelid affected. A similar study from Zaire in Africa (1962 – 1991) reported on 11 patients with ocular adnexal KS, representing an incidence of 1.25 for 10,000 cases [91]. In this study, 9 of the patients had AIDS. It has been previously pointed out that ocular KS may be the first manifestation of HIV infection [85].

External ocular KS may manifest as a mass lesion or simply as a subconjunctival hemorrhage. Interestingly, increased sludging of conjunctival blood-flow has been shown in patients with KS [92]. Epiphora (overflow of tears) due to KS of the nasolacrimal duct has been seen [93]. Far less common, KS may involve the internal structures of the eye. In one case with widely disseminated KS, tumor involving the choroid of both eyes was identified at autopsy [46]. A case of widely disseminated AIDS-associated KS localized in the patient's orbit has been previously described [94]. Despite intensive chemotherapy, progression in this patient was aggressive with a fatal outcome. While regression of conjunctival KS has been documented in some cases [95], so to has recurrence following surgical treatment and cryotherapy of ocular adnexal KS in AIDS patients [80].

## Major salivary glands

KS can involve the parenchyma and/or intraparenchymal lymph nodes of the major salivary glands [96]. Both the submandibular and parotid glands have been involved. The parotid is the only salivary gland with substantial lymphoid tissue. This explains why most case reports of KS were of intraparotid lymph nodes, particularly in the setting of AIDS [97-99]. Excluding intraparotid lymph node involvement, around 10 cases have been reported in which KS was identified infiltrating the acinar tissue of major salivary glands [100-103]. Patients with KS of their salivary glands have been reported to present clinically for evaluation primarily because of a 1 cm to 4 cm mass or swelling of the major salivary gland that was present from 1 to 70 months [103]. Findings of HHV8 DNA in saliva prompted investigators to study the presence of this virus in several salivary gland neoplasms [104]. They found that HHV8 does not appear to infect the salivary gland in HIV-seronegative patients, nor does it not seem to play a pathogenic role in non-KS vascular and epithelial salivary gland neoplasms.

## Endocrine glands

### 1. Adrenal gland

Very few detailed case reports of adrenal KS have been published, mostly in patients with AIDS [105,106]. Adrenal KS has been documented in post-mortem studies in 19% of examined patients with Classic (sporadic), 18% with African (endemic), and 17% with AIDS-related (epidemic) KS [8]. The adrenal cortex appears to be involved far more frequently than the medulla [60].

### 2. Thyroid gland

Involvement of the thyroid gland by KS is exceedingly rare, and presently only 5 cases have been reported [53,107-111]. In 2 cases the diagnosis was made at autopsy. In the other patients, the diagnosis was established by FNA. Patients have presented with a slowly enlarging asymptomatic thyroid nodule [111], as well as hypothyroidism in one case due to actual destruction of the thyroid gland by KS [108].

### 3. Pituitary gland

No report of KS involving the pituitary was found. In particular, KS of the pituitary gland was not identified in a series of 49 autopsied patients with AIDS in which the pituitary gland was specifically studied [112].

## Heart

Cardiac involvement has been reported in the various epidemiologic forms of KS. The results of autopsy studies conducted from 1959–1986 identified KS of the heart in 18% of African (endemic), 17% in AIDS-related (epidemic), and 15% of Classic (sporadic) KS [8]. Cardiac dysfunction directly due to KS involvement has not been noted [8]. In Classic KS, heart involvement has been reported to occur more likely in patients without cutaneous disease [107,113]. Surprisingly, primary cardiac KS has been found in a Haitian woman with AIDS [114]. The epicardium (subepicardial adipose tissue) appears to be more commonly involved than the myocardium or endocardium [8,115-117].

## Thoracic duct

Chylothorax is a known, but rare manifestation of KS involving the thoracic duct and adjacent mediastinal structures [118-123]. KS related chylothoraces frequently develop with concomitant upper airway KS disease [122]. Interestingly, a case of chylous ascites caused by KS has been previously documented [124]. Although KS-related chylothorax formation was initially postulated to develop due to metastatic KS to the thoracic duct [118], more recent findings suggest that this may arise due to development of in-situ KS in this region [123].

## Urinary System

Genital KS lesions are common. Infrequently, urethral meatal lesions may cause outlet obstruction and urinary retention [125,126]. However, KS of the urinary system has only been rarely reported, despite the fact that HHV8 is shed in urine from infected patients [127]. There have been three accounts of KS of the urinary bladder [128-130]. Interestingly, all three patients were renal transplant recipients. In one patient KS involved a transplanted kidney, ureter and urinary bladder [129].

## Breast

Since HHV8 can be detected in breast milk [131], it is not unexpected that KS may arise within mammary tissue. KS development on lymphedematous arms following radical mastectomy has been reported in two patients [132,133]. KS within the breast may involve breast parenchyma or intramammary lymph nodes [134]. There have been three cases reported of mammary KS [135-137]. Breast involvement has been reported without evidence of KS disease elsewhere, and also in the setting of disseminated cutaneous KS. KS can present as a small deep palpable mass or as a cutaneous lesion. KS axillary lymphatic involvement in one HIV infected patient produced lymphatic obstruction, causing a peau d'orange appearance of the breast [136].

## Wounds

Localization of KS to sites of previous iatrogenic trauma has been documented [138,139], several with underlying immunosuppression [140]. One publication describes oral KS arising after minor surgery in a transplant patient [141]. KS in this particular case developed soon (within 6 days) after the patient's trauma. A similar case of KS occurring de novo in the surgical scar of a heart transplant recipient has been published [142]. A case of primary intraosseous KS developed in the tibia of an HIV-negative 20-year-old man at the exact same site of a prior traumatic bone injury and operative scar [20]. Another osseous lesion in the mandible of a 52-year-old man with AIDS arose in the area of previously extracted teeth [23]. Moreover, there have been cases noted of KS arising in tissue grafts [143,144]. Around 10 cases of KS associated with pemphigus lesions have been described, mainly with pemphigus vulgaris and less commonly foliaceus and erythematosus variants [145,146]. KS occurring in a dermatome previously involved by herpes zoster has been shown [147], as well as primary KS due to prior radiation [148].

## Blood clots

A case of transplant-related KS restricted to the site of a previous deep venous thrombosis has been documented [149]. This 72-year-old male patient with a renal transplant had received immunosuppressant drugs including sirolimus, mycophenolate mofetil, tacrolimus and steroids. His KS developed 11 months after transplantation, in relation to deep venous thrombosis and withdrawal of sirolimus due to toxicity. Progressive withdrawal of prednisone was accompanied by full remission of this patient's KS.

## Conclusion

It is clear from the aforementioned review that KS has been reported in virtually all anatomic sites. KS involvement of other unusual sites not discussed in this chapter include the gonads (e.g. testis) [150], endometrium [46], and external auditory meatus [151]. The rarity of KS reports in uncommon regions may be due be to underreporting, asymptomatic lesions, declining number of current autopsies being performed, and/or under recognition of signs by health care providers. Many of these patients were reported to have AIDS in the pre-HAART era (<1996), with widely disseminated disease. While KS has been documented in infants as young as 6-days-old [152], KS of the placenta does not appear to have yet been reported.

As a result of HIV, a significant increase in anatomically disseminated KS cases was evident. The anatomical distribution of KS lesions also changed during the AIDS period. Soon after the onset of the AIDS epidemic, head and neck AIDS-related KS became one of the most common manifestations of AIDS [153]. In a study involving Tanzanian children, investigators noted that in approximately 12% of children KS appeared in anatomical sites that were usually not involved in cases of the pre-AIDS period, viz. the eyelid, ear, lip, face and genitalia [154]. Furthermore, researchers also reported a significant increase in upper limb cases and a decrease in lower limb and lymph node involvement [154]. The manifestations of KS have also changed considerably with the advent of HAART. As a result of HAART, a smaller proportion of KS patients appear to present with visceral disease [155]. In particular, gastrointestinal tract and pulmonary involvement are less frequent among KS-HAART patients [155]. The frequency of mucosal and lymph node involvement do not appear to be appreciably altered by HAART [155].

The occurrence of KS in any of these atypical sites may prove difficult to diagnose, particularly if patients are asymptomatic, lesions go unrecognized on routine imaging studies (e.g. osseous KS on plain x-ray films), and/or clinician's are unwary of their existence [156]. Awareness that KS can occur in any of these unusual locations may avoid potential misdiagnosis with serious consequences (e.g. spinal cord compression) and/or mismanagement (e.g. biopsy of laryngeal KS with subsequent life-threatening hemorrhage). Also, the possibility of occult HIV infection should be entertained in a young person with an unusual clinical presentation (e.g. subconjunctival hemorrhage), as KS may mimic common lesions and represent the initial presenting sign of AIDS.

In several cases, there was limited available clinical data. Pathologic verification of a diagnosis of KS by means of biopsy and/or autopsy was not available in some cases. In their review of 66 cases of reported osseous KS, the authors noted that histological proof of actual bone involvement by KS was only obtained in 53% of cases [9]. Very rarely was HHV8 demonstrated within any of these lesions. Therefore, it is plausible that some published cases may not truly represent KS. KS involvement of infrequent sites may not be considered in the differential diagnoses. In HIV infected persons, for example, brain lesions and salivary gland enlargement are frequently identified and more likely to be attributed to other non-KS causes. Hence, in the setting of HIV/AIDS, it is important to always consider KS in the differential diagnosis.

It is interesting to hypothesize why KS is rarely encountered in the aforementioned anatomic locations. Moreover, why KS involvement of sites like the bladder and breast are uncommon when HHV8 is shed in urine and breast milk, respectively, from infected patients is intriguing. A urinary protein called ANUP (antineoplastic urinary protein) has certainly been shown to inhibit KS growth in a murine model [157]. Conversely, investigators have shown that nerve growth factor, possibly related to HHV8 infection, may be involved in KS progression [158]. Moreover, KS cells secrete a neurotrophic growth factor [159]. One would expect these findings to support more frequent KS involvement of neural tissue.

It is also unclear if isolated KS lesions in some of these sites represent primary or disseminated disease. Some authors have considered the occurrence of KS in unusual locations to represent metastatic disease [54,160]. Alternatively, KS may arise in numerous sites via multicentric development. It is widely believed that since the eyes and brain are relatively devoid of lymphatic endothelium, these organs are unlikely to be sources of primary KS if KS tumor cells are indeed derived from lymphatic endothelium [160-162]. Direct histological evidence of the development of early (in-situ) KS from lymphatic vessels in the setting of chronic lymphedema has been shown [123].

KS lesions develop as a result of the following combination of factors: HHV8, altered immunity (immunosuppression), and an inflammatory/angiogenic milieu. This may explain why, in the appropriate clinical setting, KS develops in traumatized tissue or pemphigus lesions [145]. Some authors believe that post-traumatic KS lesions represent the Koebner phenomenon (the appearance of a skin lesion as a result of trauma) [141,147]. The Koebner phenomenon has been reported by several authors to develop in Classic, AIDS-related and transplant-associated KS [163,164]. Although still controversial, pemphigus has been associated with HHV8 [165]. Trauma may precipitate inflammatory changes that recruit HHV8 to the injured site. Finally, specific inflammatory cytokines like interleukin-6, shown to be increased in both serum and blister fluid of pemphigus patients [166], are essential for KS tumorigenesis.

## Competing interests

The authors declare that they have no competing interests.

## Authors' contributions

LP and BJD contributed equally to all aspects of this paper.

## Pre-publication history

The pre-publication history for this paper can be accessed here:


